# The Role of Community in Understanding Involvement in Community Energy Initiatives

**DOI:** 10.3389/fpsyg.2021.775752

**Published:** 2022-02-09

**Authors:** Fleur Goedkoop, Daniel Sloot, Lise Jans, Jacob Dijkstra, Andreas Flache, Linda Steg

**Affiliations:** ^1^Department of Psychology, Faculty of Behavioural and Social Sciences, University of Groningen, Groningen, Netherlands; ^2^Department of Sociology, Faculty of Behavioural and Social Sciences, University of Groningen, Groningen, Netherlands; ^3^Chair of Energy Economics, Institute for Industrial Production (IIP), Karlsruhe Institute of Technology, Karlsruhe, Germany

**Keywords:** community energy initiatives, community involvement, community identification, interpersonal contact, personal motivation

## Abstract

Community energy initiatives are set up by volunteers in local communities to promote sustainable energy behaviors and help to facilitate a sustainable energy transition. A key question is what motivates people to be involved in such initiatives. We propose that next to a stronger personal motivation for sustainable energy, people’s perception that their community is motivated to engage in sustainable energy and their involvement in the community (i.e., community identification and interpersonal contact) may affect their initiative involvement. We tested this proposition with a questionnaire study among inhabitants of seven local communities (*N* = 439). Results suggested that community factors are uniquely related to initiative involvement (willingness to actively participate and attendance of an initiative meeting) next to personal sustainable energy motivations. In particular, stronger community identification and more interpersonal contact with other community members increased the likelihood that people become involved in a community initiative, but the perception of the sustainable energy motivation of one’s community was not uniquely related to initiative involvement. We discuss theoretical and practical implications of these findings.

## Introduction

Community energy initiatives (CEIs) could help in promoting a sustainable energy transition (Middlemiss and Parrish, 2010; Intergovernmental Panel on Climate Change [IPCC], 2018; Creamer et al., 2019). Typically, a CEI is initiated by community members aiming to promote more sustainable energy behaviors in their local community. Activities include the (collective) purchase of solar cells and better home insulation, producing renewable energy locally, encouraging energy saving at home, or even achieving energy neutrality of the entire community. Research suggests that those involved in CEIs generally behave more sustainably (Middlemiss, 2011; Sloot et al., 2018). Yet, to achieve their full potential, it is key that a sufficient number of community members become involved in a CEI. This raises the question of what motivates people’s involvement in a CEI.

Research points to personal pro-environmental motivations as a key predictor of whether and to what extent someone engages in different kinds of pro-environmental behaviors, including involvement in CEIs (Stern, 2000; Steg et al., 2015; Van der Werff and Steg, 2016; Sloot et al., 2018). However, involvement in CEIs concerns more than engaging in a specific pro-environmental behavior. It allows individuals to meet and connect with other community members and pursue common goals, which can motivate people’s involvement in CEIs, too (Sloot et al., 2019). In this regard, CEIs share similarities with forms of collective action such as social movements (Bell et al., 2005; see also Simon and Klandermans, 2001). However, whereas social movements typically aim to change the system or their group’s position relative to other groups, CEIs have the goal to change their own community (e.g., Sloot et al., 2017). CEIs can be seen as initiatives for providing impure collective goods (see Olson, 1965) which potentially generate both private, individual benefits, and collective benefits for CEI members, the whole community and society at large (e.g., Bauwens, 2017). This suggests that next to personal pro-environmental motivations, community factors could play a unique role in explaining initiative involvement. This would imply potential novel pathways to promoting pro-environmental behavior through targeting relevant community factors. So far, little is known about which community factors, if any, encourage CEI involvement. We address this gap by examining the role of two community factors in predicting CEI involvement: the perceived sustainable energy motivation of the community, and individuals’ level of involvement in the local community. Specifically, we examine the extent to which individuals feel psychologically involved in their community, as reflected in their identification with the community, and the extent to which they have actual interpersonal contact with other community members. By studying the relative importance of each of these factors in explaining CEI involvement, we extend the limited, and mostly qualitative, research that examined the extent to which each of these community factors separately affects initiative involvement (Hoffman and High-Pippert, 2010; Bomberg and McEwen, 2012; Rees and Bamberg, 2014; Bauwens, 2016; Biddau et al., 2016; Kalkbrenner and Roosen, 2016). Notably, we take into account people’s personal sustainable energy motivation to examine the unique influence of community factors over and above this personal motivation. We elaborate on these factors below.

## Pro-Environmental Motivations Underlying CEI Involvement

### Personal Motivations

Individuals are more likely to become involved in a CEI when they have a stronger personal pro-environmental motivation (Hoffman and High-Pippert, 2010; Sloot et al., 2018, 2019). This may be reflected in more general motivations to protect the environment and to act in an environmentally friendly way (e.g., biospheric values and environmental self-identity; e.g., Van der Werff and Steg, 2016). Yet, personal pro-environmental motivations have been shown to be more predictive of initiative involvement when they are specific to the behavior targeted by the initiative in question, such as the extent to which people find sustainable energy behavior personally important (Sloot et al., 2018). Hence, we expect that the more people find it important to engage in sustainable energy behavior, the more likely they are to be involved in CEIs.

### Perceived Community Motivations

Next to personal motivations, perceptions of what motivates relevant others and groups can influence individuals’ thoughts, feelings, or actions (Cialdini et al., 1990; Turner, 1991; Klandermans, 2004; Christakis and Fowler, 2009; Peattie, 2010; Dunlap and Brulle, 2015), and whether they behave pro-environmentally or not (Axsen and Kurani, 2012; Fielding and Hornsey, 2016; Mignon and Bergek, 2016; Fritsche et al., 2018). Different strands of literature have investigated different indicators of group motivations, such as social norms (e.g., Bicchieri, 1990; Cialdini et al., 1990), second-order normative beliefs (e.g., Jachimowicz et al., 2018), or group values (Bouman et al., 2020). People may be motivated to act in line with perceived group motivations because they perceive these actions as effective, normal, or appropriate in a given situation, because they want to avoid social sanctions from others, and/or because they internalize these group goals as their own (see Turner, 1991; Fritsche et al., 2018). One’s local community can be a relevant social group, affecting people’s behaviors (e.g., Nolan et al., 2008; Jachimowicz et al., 2018; Bouman and Steg, 2019; Jans, 2021). As such, we reason that next to individuals’ personal sustainable energy motivation, involvement in a community energy initiative is more likely when people believe that other community members find it important to engage in sustainable energy behavior. We denote this community factor community sustainable energy motivation.

### Community Involvement

People’s CEI involvement may not only be influenced by the motivations they perceive other community members to hold, but also by the extent to which individuals are involved in their community. We examine two indicators of community involvement. First, individuals can feel more or less psychologically involved in their community, as reflected in their level of identification with the community (Leach et al., 2008; Postmes et al., 2013; see also Obst et al., 2002, on related concepts of sense of community in the field of community psychology). Second, individuals can have more or less actual interpersonal contact with others in the community, which reflects the personal bonds with other community members, created through social interaction (Lindenberg, 1997; Deaux and Martin, 2003; Moody and White, 2003; Völker and Flap, 2007; Easterbrook and Vignoles, 2012). While some initial research has examined the role of community identification for initiative involvement, interpersonal contact has not been integrated into these models (e.g., Rees and Bamberg, 2014; Bamberg et al., 2015; Kalkbrenner and Roosen, 2016). Identifying with one’s community or having interpersonal contact with community members reflect different conceptualizations of community involvement (cf. Stets and Burke, 2000; Deaux and Martin, 2003) and we expect both factors to be important for participation in a CEI, albeit through different processes.

First, both indicators of community involvement may affect the extent to which the perceived sustainable energy motivation of other community members affects involvement in CEIs. Community identification affects the extent to which community members internalize their local community’s motivations and, consequently, behave in line with these community motivations (Masson and Fritsche, 2014; Fielding and Hornsey, 2016; Fritsche et al., 2018). Interpersonal contact increases possibilities for effective social control (Granovetter, 1985; Coleman, 1990) as it gives a person a larger stake in living up to other people’s expectations, and it provides more channels for social influence between people (Axsen and Kurani, 2012). As such, people are generally more likely to act in line with community motivations in communities with many ongoing interpersonal contacts (Festinger, 1954; Cartwright and Zander, 1968; Bicchieri, 1990). Thus, we propose that the more people identify with their community and the more interpersonal contacts they have with other community members, the more likely it is that the perceived sustainable energy motivations of other community members drives their CEI involvement.

Second, both indicators of involvement in one’s community may affect CEI involvement directly, independent of any community sustainable energy motivations, as being strongly involved in one’s community entails that community members may be generally more motivated to cooperate and engage in collective actions with other community members (Austin and Worchel, 1986; Pretty and Ward, 2001; Haslam, 2004; Tindall, 2008; see also Klandermans, 2004, on related models of collective action). To date, it is unclear to what extent identification with the community and interpersonal contact with others within a community can uniquely contribute to explaining CEI involvement. Although one can exist without the other, we propose that both concepts of community involvement can increase the likelihood that people become involved in a CEI and additionally enhance the extent to which one acts in line with the community’s sustainable energy motivations by becoming involved.

### Current Research

To test our hypotheses, we conducted a questionnaire study in seven local communities in the Netherlands in which a CEI had recently started aiming to make their local community energy-neutral within the next 10 years, comprising six villages and one city neighborhood. The communities varied in aspects like their location, their size (between 395 and 2125 households) and housing stock. The initiatives followed different strategies to achieve the goal of energy neutrality, generally aiming at generating renewable energy via for instance collective PV solar installations, next to raising awareness regarding energy saving and renewable energy alternatives. All of these CEIs were supported by the Dutch foundation Stichting Samen Energieneutraal (translated: Foundation Together Toward Energy Neutrality), which provided advice and functioned as an umbrella network organization for all the local initiatives.

Since people could not formally sign up as initiative members yet, we examined two indicators of CEI involvement, namely people’s willingness to actively participate in the initiative in terms of financially investing or volunteering in the initiative (e.g., helping with organizing events, being part of smaller working groups), and actual attendance of an initiative meeting. Willingness to participate reflects an early stage in one’s decision process to join a CEI in the future, but people may not follow up on their expressed willingness by taking actual steps of being involved in the initiative. Initiative meeting attendance complements this measurement of initiative involvement by capturing an actual (self-reported) behavior (although of course not shedding light on people’s actual membership or continuous involvement).

**FIGURE 1:**
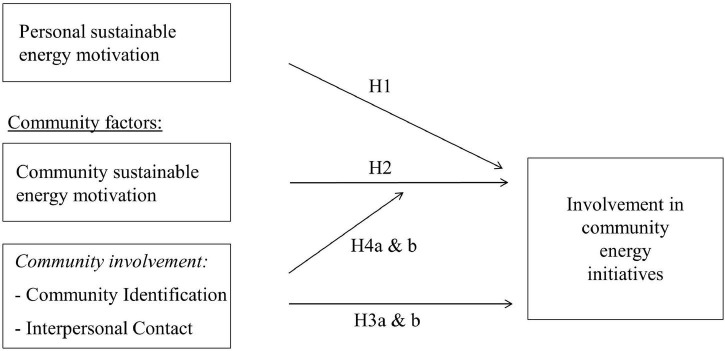
Conceptual model.

In sum, first, we expected a stronger personal sustainable energy motivation to be related to increased initiative involvement (H1; see Figure 1). Second, we expected a stronger perceived community sustainable energy motivation to increase the likelihood of initiative involvement, over and above the effects of personal sustainable energy motivations (H2). Third, we hypothesized that stronger community involvement, in terms of a higher level of identification with the community (H3a) and more interpersonal contact with others in the community (H3b), uniquely promotes initiative involvement. Fourth, we expected the relationship between community sustainable energy motivation and initiative involvement to be stronger among individuals who are strongly involved in their community, both in terms of a higher level of identification with their community (H4a) and more interpersonal contact with other community members (H4b). As community identification and interpersonal contact have previously been examined by relatively independent bodies of literature, we tested their respective influence in separate (i.e., parallel) models first, before examining their relationship with initiative involvement simultaneously in one model.

## Materials and Methods

### Sample and Procedure

The data presented in this paper were collected in the context of a large project on community energy initiatives^1^. We collected data in seven local communities in the Netherlands in which a CEI had recently started. Data was collected within these communities between 2015 and 2018, after an information evening about the initiatives had been organized. First, an information letter about the upcoming study was sent to community members (8,727 households in total; administered in Dutch), additionally containing a short survey with a request for participation in the main questionnaire and a prepaid response envelope. We asked one adult resident per household to fill out the survey, indicate whether they were willing to participate in the study, and send it back to us. Additionally, people could indicate whether they would like to receive an email with a link to an online questionnaire or request a paper version of the questionnaire that would be sent to them via regular mail; in this case, they needed to fill out their address details. In addition, a total of 600 questionnaires were delivered door-to-door to a random sample of initially approached households who had not responded to our first request for participation. The contact details of the participants were always kept separate from the actual questionnaire data, so that anonymity of the data was ensured. In three of the seven communities, the variables for the present study were asked in a short follow-up questionnaire sent shortly after participants had filled out the main questionnaire, as the original questionnaire in these communities had not included the relevant variables for this study. Households were only re-approached if they had indicated on the main questionnaire to be willing to be involved in any follow-up questionnaires, which all but 19 did. The total number of households approached for the main (or where applicable the follow-up) questionnaire was 1,696 (19% out of the initial 8,727 residents approached), of which 550 completed the questionnaire (response rate: 32%, ranging from 13% to 40% across communities). In total, 52% of the respondents filled out a questionnaire online and 48% filled out the questionnaire on paper.

To facilitate comparisons between the different analysis steps, the sample is limited to respondents who had non-missing values on all variables used in this study, resulting in 487 cases. Furthermore, we removed all respondents who indicated to be initiators of the initiatives (as they were already part of the initiative), 48 in total, which reduced the sample to 439 respondents. Of these, 63% were male and 37% female, with a mean age of 59.56 (*SD* = 14.03). Most respondents had either completed secondary vocational education or training (38.7%) or higher education (47.2%). The median household income level was 2,000-2,999 euros net per month (ranging from less than a 1,000 euros net per month to 4,000 or more).

### Measures

In the order listed below, the questionnaire measured personal sustainable energy motivation, community sustainable energy motivation, identification with the community (items across these three measures were all shuffled), interpersonal contact with community members, as well as the two dependent variables willingness to participate in the community energy initiative and initiative meeting attendance.

***Personal sustainable energy motivation*** was assessed via three items: “I find it important to be conscious about my energy behavior,” “I find it important to reduce my energy consumption,” and “I find it important to use sustainable energy” (Sloot et al., 2018). Answers were provided on a 7-point scale ranging from (1) completely disagree to (7) completely agree; we computed mean scores on these items (α = 0.79; *M* = 5.50, *SD* = 1.01).

***Community sustainable energy motivation*** was measured using the same statements to capture personal sustainable energy motivation, with “I” replaced by “inhabitants of my neighborhood.” Mean scores were computed on these items (α = 0.92; *M* = 4.30, *SD* = 1.11).

***Identification with the community*** was measured using the following four statements: ‘‘I identify with my neighborhood^2^,” “I feel committed to my neighborhood,” “I am glad to be a resident of my neighborhood,” and “Being a resident of my neighborhood is an important part of how I see myself” (Postmes et al., 2013). Answers were provided on a 7-point scale ranging from (1) completely disagree to (7) completely agree (α = 0.89; *M* = 4.66, *SD* = 1.32). We computed mean scores on these items; at least three items needed to be answered in order to obtain a value on the scale.

***Interpersonal contact with community members*** was measured using the following three questions: “How often do your neighbors visit you?” “How often do you visit your neighbors?” “How often do you participate in activities in your community together with neighbors?” (adjusted from Dykstra et al., 2005). Answers were provided on a 5-point scale ranging from (0) never; (1) almost never; (2) a couple of times per year; (3) a couple of times per month; (4) a couple of times per week. Mean scores on these items were computed (α = 0.84; *M* = 2.06, *SD* = 0.88).

***Willingness to participate in the community energy initiative.*** We first briefly informed respondents about the local initiative via the following statement: “The following questions are concerned with energy saving and production via local sustainable energy initiatives. The research focuses specifically on an initiative, initiated by community members, that started recently in this community named [add name].” Willingness to participate was then measured via two questions: “Do you want to volunteer in this community energy initiative” and “Do you want to financially invest in this community energy initiative?” Answers could be (0) no; (1) maybe; (2) yes; (3) already participating or already financially contributing; this last category was excluded from the analyses as only the initiative initiators (*N* = 48) were “already participating” at the point of data collection. Both questions were positively correlated (*r*_*s*_ = 0.53; *p* < 0.01). As answering either one of these questions indicates a willingness to become involved, we combined these two items by using a maximum score, that is, using the highest score on either of the two questions for each respondent (instead of a mean score, which would be inconsistent with the categorical ordinal type of this scale; [no] 28.4%; [maybe] 64.3%; [yes] 7.3%)^3^.

***Initiative meeting attendance.*** Respondents indicated whether or not they had attended the information meeting about the initiative that had been organized prior to the data collection. Respondents could respond with (0) no (81.8%) or (1) yes (18.2%).

### Data Analysis

All data was analyzed using R (R Core Team, 2017). We conducted our analyses separately for the two indicators of initiative involvement, namely willingness to participate and initiative meeting attendance. We first examined the bivariate correlations between all variables. Next, we examined the relationships between the predictor variables and the two indicators of initiative involvement through a proportional odds model (for willingness to participate) and logistic regression analyses (for initiative meeting attendance), respectively. To avoid multicollinearity problems in the interaction models and facilitate interpretation of the effects, all continuous predictor variables were mean-centered for the regression analyses (Afshartous and Preston, 2011). As the proportional odds assumption (Brant, 1990) in ordered regression models of equal slopes for all transition levels of the dependent variable (willingness to participate) was violated for community sustainable energy motivation, identification with the community, and interpersonal contact, we used a partial proportional odds model. This model allows for these factors to have different estimates for different levels of willingness to participate (Yee, 2010, 2015). Thresholds, one for each transition of willingness to participate (maybe or yes versus not participating and yes versus maybe or not participating) are comparable to the constant term in binary logistic regression. All coefficients are shown in log-odds^4^. All models were controlled for the interdependence of data within communities using community-fixed effects^5^.

We used a hierarchical regression approach in order to examine the unique role of each predictor variable in explaining CEI involvement. In the first (baseline) model, we only included personal sustainable energy motivation (Step 1) to examine its relation with initiative involvement. Next, we added perceived community sustainable energy motivation to test for its main effect on CEI involvement (Step 2). In the third step, we added identification with the community as an additional predictor to this model (Step 3a) to test for its main effect, and afterward added the interaction term between community sustainable energy motivation and identification with the community (Step 4a). In a parallel model, we added interpersonal contact to the model estimated in the previous Step 2 to test its main effect on initiative involvement (Step 3b) and afterward added its interaction with community sustainable energy motivation (Step 4b). Finally, we estimated the full model in which we added both main effects of identification with the community and interpersonal contact simultaneously (Step 3c), and both interaction terms (step 4c), to examine the relative strength of identification with the community and interpersonal contact in explaining initiative involvement.

## Results

Bivariate correlations (Table 1) showed that personal sustainable energy motivation was positively related to willingness to participate but not related to meeting attendance. The three community factors (community sustainable energy motivation, identification with the community and interpersonal contact) correlated positively with each other, and, as expected, were all positively related to both indicators of CEI involvement. We note that identification with the community and interpersonal contact are significantly correlated but also different from each other, supporting our proposition that it is important to consider them separately. People who attended an initiative meeting indicated to be somewhat more willing to participate in the initiative, yet these two variables only showed a weak to moderate correlation.

**TABLE 1:** Correlations between all dependent and independent variables used in the analyses.

	(1)	(2)	(3)	(4)	(5)
(1) Personal sustainable energy motivation					
(2) Community sustainable energy motivation	0.21***				
(3) Identification with the community	0.11*	0.52***			
(4) Interpersonal contact with community members	0.10*	0.34***	0.54***		
(5) Willingness to participate	0.15**	0.12*	0.16**	0.11*	
(6) Initiative meeting attendance	0.07	0.11*	0.14**	0.16**	0.21**

*p < 0.05, **p < 0.01, ***p < 0.001. Correlations between the two outcome variables and the predictors were computed using the Spearman rank coefficient; the intercorrelation between the two outcome variables is assessed via Cramer’s V (see Supplementary Appendix 1 for chi square test).

### Willingness to Participate

The first regression model showed that a stronger personal sustainable energy motivation was related to a higher willingness to participate in the CEI (Table 2; Step 1). When adding community sustainable energy motivation to the model in the second step, we found that a stronger community sustainable energy motivation was significantly related to a higher willingness to participate; specifically, it explained whether people would or maybe would participate (versus not; Table 2; Step 2).

**TABLE 2:** Partial proportional odds model of willingness to participate in a community energy initiatives on personal sustainable energy motivations, community sustainable energy motivation and community involvement.

	Step 1		Step 2		Step 3a		Step 3b		Step 4a		Step 4b		Step 3c		Step 4c	
	Estimate	*p*	Estimate	*p*	Estimate	*p*	Estimate	*p*	Estimate	*p*	Estimate	*p*	Estimate	*p*	Estimate	*p*
Threshold 1	–1.28*	0.017	−1.17*	0.028	–1.28*	0.016	–1.22*	0.022	–1.31*	0.015	–1.22*	0.022	–1.23*	0.020	–1.24*	0.021
Threshold 2	2.49***	<0.001	2.65***	<0.001	2.57***	<0.001	2.67***	<0.001	2.46***	<0.001	2.67***	<0.001	2.66***	<0.001	2.61***	<0.001
Personal motivation	0.37***	<0.001	0.32**	0.002	0.34**	0.002	0.32**	0.003	0.36**	0.001	0.34**	0.002	0.35**	0.002	0.36**	0.001
Community motivation [No vs. Maybe-Yes] [No-Maybe vs. Yes]			0.24* 0.28*	0.037 0.012	0.05 0.23	0.689 0.273	0.21 0.16	0.072 0.422	–0.04 0.41	0.775 0.062	0.19 0.28	0.123 0.194	0.06 0.21	0.653 0.341	0.04 0.45*	0.768 0.049
Identification with the com. [No vs. Maybe-Yes] [No-Maybe vs. Yes]					0.31** 0.09	0.003 0.633			0.30** 0.18	0.004 0.335			0.36** –0.10	0.004 0.632	0.34** 0.02	0.005 0.934
Interpersonal contact [No vs. Maybe-Yes] [No-Maybe vs. Yes]							0.13 0.49*	0.366 0.049			0.13 0.64*	0.373 0.014	–0.10 0.53	0.558 0.050	–0.10 0.66*	0.536 0.027
Community motivation × Identification with the com. [No vs. Maybe-Yes] [No-Maybe vs. Yes]									–0.03 –0.43*	0.574 0.013					–0.03 –0.41*	0.765 0.038
Community motivation × Interpersonal contact [No vs. Maybe-Yes] [No-Maybe vs. Yes]											–0.06 –0.41	0.585 0.067			–0.03 –0.18	0.822 0.532
AIC	657.63		655.80		651.01		655.60		649.04		655.88		648.87		651.26	

Unstandardized coefficients and standard errors. *p < 0.05, **p < 0.01, ***p < 0.001, N = 439. All results are shown in log odds and controlled for community fixed effects, gender, and education.

Next, when taking personal and community sustainable energy motivation into account, we found that stronger identification with the community was related to higher willingness to participate (Table 2; Step 3a). Notably, stronger identification with the community explained whether people would or maybe would participate (versus not). After adding community identification to the model, the relation between community sustainable energy motivation and willingness to participate in a CEI became non-significant.

We then tested whether the relationship between community sustainable energy motivation and willingness to participate was stronger when people identified more strongly with their community (Table 2; Step 4a). Yet, opposite to our expectation, we found a negative interaction effect between community sustainable energy motivation and identification with the community on willingness to participate (for the threshold from willing to participating versus not or maybe; all other relationships remained as in the previous model). Specifically, simple slopes (specific to the relevant threshold from willing to participate versus not or maybe willing to participate) suggested that community sustainable energy motivation was more strongly related to willingness to participate among respondents who less strongly identified with their community. Specifically, among those weakly identified with the community (-1 *SD*), community sustainable energy motivation was positively related to willingness to participate (*b* = 0.95; *p* < 0.001) whereas for strongly identified community members (+ 1 *SD*), this relationship was not significant (*b* = −0.15; *p* = 0.609; see Supplementary Appendix 2 for figures displaying the simple slopes).

In parallel hierarchical regression models, we examined the effects of interpersonal contact, instead of identification on CEI involvement, next to personal and community sustainable energy motivation. Stronger interpersonal contact was associated with a higher willingness to participate, explaining whether people would participate (versus not or maybe). Besides, the effect of personal sustainable energy motivation remained significant, while the effect of community sustainable energy motivation was no longer significant (see Table 2; Step 3b). Yet, contrary to our expectations, we did not find a significant interaction effect between community sustainable energy motivation and interpersonal contact on willingness to participate (Table 2; Step 4b).

Lastly, we examined the extent to which identification with the community and interpersonal contact were uniquely related to willingness to participate (Table 2; Step 3c and 4c). Both identification with the community and interpersonal contact were uniquely positively related to willingness to participate (Table 2; Step 3c), yet interpersonal contact became only marginally significant (*p* = 0.050)^6^. Specifically, stronger identification with the community particularly seemed to explain whether people would or maybe would participate (versus not). More interpersonal contact seemed to mainly explain whether people would participate (versus not or maybe). We note that while the bivariate correlations showed these concepts to be significantly correlated, Variance Inflation Factor (VIF) scores indicated no concern for multicollinearity (the maximum VIF value being 1.82) which is well below the commonly used critical threshold of 10. The combined model, without the combined interaction effects, provided the best fit with the data based on the AIC criterion, compared to the other models, suggesting that community sustainable energy motivation and community involvement are mostly independently related to willingness to participate, rather than interacting with each other.

Adding the interaction terms between community sustainable energy motivation and identification with the community and community sustainable energy motivation and interpersonal contact, respectively (Table 2; Step 4c), yielded similar results compared to the previous models (see Table 2; Step 4a), showing the same negative interaction effect between identification with the community and community sustainable energy motivation on willingness to participate (with similar simple slopes) we found before.

### Initiative Meeting Attendance

In contrast to willingness to participate, a stronger personal sustainable energy motivation was not significantly related to an increased likelihood of attending an initiative meeting (Table 3; Step 1). Yet, similar to our results for willingness to participate, stronger community sustainable energy motivation increased the likelihood of initiative meeting attendance (Table 3; Step 2) and when taking personal and community sustainable energy motivation into account, we found that stronger identification was also related to a higher likelihood of attending the initiative meeting (Table 3; Step 3a). Again, adding community identification to the model, the relation between community sustainable energy motivation and CEI involvement became non-significant. The interaction between community sustainable energy motivation and identification had no significant effect on initiative meeting attendance, and only the effect of identification with the community remained significant in this model (Table 3; Step 4a).

**TABLE 3:** Binomial logistic regression of initiative meeting attendance on personal sustainable energy motivations, community sustainable energy motivation and community involvement. Unstandardized coefficients and standard errors.

	Step 1		Step 2		Step 3a		Step 3b		Step 4a		Step 4b		Step 3c		Step 4c	
	Estimate	*p*	Estimate	< *cps*:*it* > *p* < /*cps*:*it* >	Estimate	*p*	Estimate	*p*	Estimate	*p*	Estimate	*p*	Estimate	*p*	Estimate	*p*
Intercept	−1.76*	0.014	−1.97**	0.008	−1.82**	0.016	−1.98**	0.009	−1.84*	0.015	−1.98**	0.009	−1.86*	0.014	−1.84*	0.016
Personal motivation	0.25	0.105	0.19	0.228	0.17	0.293	0.17	0.292	0.16	0.326	0.15	0.363	0.17	0.317	0.15	0.363
Community motivation			0.43**	0.003	0.22	0.206	0.32*	0.039	0.21	0.210	0.32*	0.041	0.21	0.227	0.21	0.235
Identification with the com.					0.41**	0.005			0.39**	0.005			0.26	0.089	0.30	0.076
Interpersonal contact							0.56**	0.003			0.54**	0.005	0.40	0.056	0.36	0.102
Community motivation × Identification with the com.									–0.02	0.827					–0.05	0.683
Community motivation × Interpersonal contact											0.13	0.387			0.19	0.340
AIC	352.15		345.09		338.54		337.91		340.50		339.19		336.91		339.92	

*p < 0.05, **p < 0.01, *** p < 0.001. N = 439. All results are shown in log odds and controlled for community fixed effects, gender, and education.

Findings on the effects of interpersonal contact show similar patterns compared to willingness to participate: more interpersonal contact was significantly associated with a greater likelihood to attend an initiative meeting. Yet, again contrary to our expectations, we did not find a significant interaction effect between community sustainable energy motivation and interpersonal contact (Table 3; Step 4b).

Lastly, we found that both indicators of community involvement were not significantly related to initiative meeting attendance when included in the same model (Table 3; Step 3c). Interestingly, this model provided the best fit to the data, which could be because the effects of community involvement were both marginally significant (*p* = 0.089; *p* = 0.056). Moreover, in the final model both interaction effects were not statistically significantly related to initiative meeting attendance (Table 3; Step 4c).

### Summary

In sum, personal sustainable energy motivation was significantly associated with people’s willingness to participate but not with attending an information evening, partly supporting H1. In addition, community sustainable energy motivation seems to be only partly related to initiative involvement (that is, in some of the tested models), which does not lend much support to H2. Both a stronger identification with the community and more interpersonal contact with community members were generally positively associated with people’s willingness to participate in the initiative and meeting attendance, although both indicators of community involvement differed in the particular transition they explained in people’s willingness to participate. Yet, in the final models, both indicators of community involvement (i.e., community identification and interpersonal contact) were uniquely related to willingness to participate but not to meeting attendance, thus only partly supporting H3a and H3b. We found no support for our hypothesis that a stronger community involvement strengthens the relationship between community sustainable energy motivation and willingness to participate (H4a and H4b). If anything, results indicate that community sustainable energy motivation particularly explains willingness to participate when people do not strongly identify with their community.

## Discussion

This paper addresses the question of why people become involved in CEIs by investigating the relationship between different community factors and initiative involvement, taking into account people’s personal sustainable energy motivations. In particular, we examined the role of community sustainable energy motivation and two indicators of community involvement—the level of identification with the community and the level of interpersonal contact with other community members—in explaining people’s willingness to actively participate in a CEI and their attendance of an initiative meeting. We found that a stronger personal sustainable energy motivation was significantly related to higher willingness to participate in the initiative, but not to attending an initiative meeting. Our results further indicated that community factors generally play a role in explaining both indicators of community involvement when taking into account one’s personal sustainable energy motivation, though different community factors appeared to be important across different models. First, perceiving the community to be more motivated to engage in sustainable energy behavior particularly increased the likelihood to become involved in a CEI when identification with the community and interpersonal contact were not considered, which lends only partial support to our second hypothesis. Next, we found that both identification with the community and interpersonal contact were positively related to willingness to participate in a CEI and attending an initiative meeting when accounting for personal motivation and community sustainable energy motivation. Interestingly, while also being quite strongly related to one another, both community identification and interpersonal contact were generally significantly related to different levels of willingness to participate when considered simultaneously, however they were both not uniquely related to initiative meeting attendance (partly supporting H3). It might be that meeting attendance (reflecting an actual behavior) is less well explained by the examined factors than willingness to participate as time constraints can be a barrier (i.e., people could simply not have been able to attend the meeting at the fixed date even though they were willing to participate). In addition, the relationship between community sustainable energy motivation and initiative involvement did not seem to be stronger among residents who are strongly involved in their community, even pointing in the opposite direction in the case of willingness to join (not supporting H4). In summary, our results indicate that different community factors, in particular those regarding the level of community involvement, uniquely contribute to the explanation of initiative involvement over and above people’s pro-environmental motivations, with some differences across the two indicators of initiative involvement.

### Theoretical Implications

Our results suggest that community factors play a role in explaining why people decide to become involved in CEIs. These findings extend previous preliminary findings on the role of community factors in guiding individuals’ involvement in CEIs (Bamberg et al., 2015; Kalkbrenner and Roosen, 2016). Specifically, previous studies mainly conceptualized community factors as community identification rather than examining other potentially relevant community factors, and also did not account for personal pro-environmental motivations, which leaves open whether and which community factors can uniquely contribute to explaining initiative involvement. We extend previous literature by examining multiple community factors simultaneously, showing that distinct community factors are uniquely related to involvement in CEIs, but at the same time not all community factors we considered were equally relevant in explaining different types of CEI involvement. Specifically, we do not find that individuals’ perception of the community sustainable energy motivation increases initiative involvement in addition to the two indicators of community involvement (i.e., identification with the community and interpersonal contact with other community members). This finding stands in contrast to research highlighting the importance of people’s perception that their community finds engaging in sustainable behavior important for their own behavior (Fritsche et al., 2018; Jachimowicz et al., 2018). First, this might be explained by the moderate to high correlations between community sustainable energy motivation and the two indicators of community involvement, which makes it more difficult to detect unique effects of community sustainable energy motivation when both, or all three, variables are included. Second, initiative involvement may be primarily predicted by people’s involvement in their community in general and not by specific pro-environmental motivations the community is perceived to hold (such as community sustainable energy motivations). This is in line with our reasoning that involvement in CEIs may bring about additional benefits: next to the opportunity to (jointly) pursue a pro-environmental goal (e.g., engaging in sustainable energy behavior), CEIs allow people in the community to meet and connect with each other. The unique character of initiative involvement being inherently social may explain the important role of community involvement in explaining initiative involvement, independent of the specific sustainable goals and motivations individuals may personally have or perceive their community to have.

Contrary to our theoretical reasoning and previous findings (Masson and Fritsche, 2014; Dietz and Whitley, 2018), we do not find that identification with the community and interpersonal contact within the community enhance the effect of community sustainable energy motivation on initiative involvement. One explanation could be that people who more strongly identify with the community have already internalized the community sustainable energy motivation (Turner, 1991), leaving little room for community sustainable energy motivation to still have a unique effect on initiative involvement (next to personal motivation), whereas for people who do not strongly identify with their community, the perception that other community members support sustainable energy could be more relevant in explaining initiative involvement^7^. In that case, we can speculate that people would adhere to the community’s sustainable energy motivation out of social pressure rather than identification with the community. Yet, from our results it seems that sustainable energy motivation and community involvement may operate independently, indicating that people may become involved in community energy initiatives either because of the (pro-environmental) initiative goals (an environmental route) or because of their involvement in their community (a social, or communal, route). Interestingly, though both may be important, we find community involvement to be the more consistent predictor of initiative involvement relative to environmental group motivations.

Importantly, we generally find both identification with the community and interpersonal contact to be positively related to involvement in the CEI. In other words, people may join CEIs because they *feel* attached to their community as a whole and because they actually *engage* in interaction with other members of the community. These findings support the relevance of one’s attachment to the group (e.g., Fritsche et al., 2018; Jans et al., 2018) and one’s interpersonal contact with other group members (e.g., Axsen and Kurani, 2012) in predicting a broad range of sustainable behaviors and collective action more generally (e.g., Briet et al., 1987; Simon et al., 1998; Mummendey et al., 1999; Simon and Klandermans, 2001; Stürmer and Simon, 2004; Mazzoni and Cicognani, 2013). These findings are also in line with findings from community psychology highlighting the importance of a sense of community for involvement in community organizations and participation in the community more generally (e.g., Obst et al., 2002). Thus, our findings support the idea that initiative involvement is inherently social and thus particularly influenced by the social context of the local community. Yet, whereas the pattern of results for community identification and interpersonal contact appears rather similar, the mechanisms through which they operate are likely different. For example, as theorized by the social identity approach (Tajfel and Turner, 1979; Turner, 1991), community identification might affect initiative involvement because highly identified people internalize the community goals as their own goals, whereas interpersonal contact has been argued to result in social influence via for example social control (Granovetter, 1985; Coleman, 1990), or both. Future research could investigate the different mechanisms through which identification with the community and interpersonal contact influence initiative involvement, and the differential effects they may therefore have. Moreover, both concepts might also mutually reinforce one another and be *causally* related to each other. For example, identities may form through interaction and communication over time (Postmes et al., 2005; Jans et al., 2015; Thomas et al., 2016) or alternatively, identities create opportunities and constraints for interaction (Deaux and Martin, 2003). While we find unique effects of both factors, further research could test if identification with the community might increase as a result of contact people engage in or vice versa, by examining the drivers of initiative involvement in a longitudinal and experimental design.

In sum, our results imply that involvement in one’s local community might motivate those people not particularly interested in sustainable behavior. Specifically, our research suggests individuals may not (only) be motivated to become involved in a CEI because of its primary cause but for communal reasons: identifying with one’s local community and being in contact with those in the community. More attention should thus be given to the role of community involvement in explaining involvement in CEIs, which might stimulate a multiplicity of sustainability-related community behaviors (cf. Sloot et al., 2019, 2021).

### Limitations and Future Directions

We conducted our research in real-life local communities in which a CEI was being set up and our findings thus have a high ecological validity. We extend previous research by looking at residents who are actually facing the (future) choice to become involved in their local CEI, instead of looking at already involved members or mere hypothetical interest in community energy among the general public where people do not face the choice to become involved in an actual local initiative in their own community (cf. Kalkbrenner and Roosen, 2016). However, due to the early stages of initiative development, we could not measure actual initiative involvement, leaving it open to what extent our indicators of initiative involvement are predictive of actual and continuous initiative involvement.

In addition, our insights are based on correlational evidence, thus not allowing causal conclusions. Yet, since our study was conducted prior to actual involvement, interpersonal contact and community identification were not a result of participation in the initiative (though this does of course not imply causality).

Moreover, we measured community sustainable energy motivation, identification with the community, and interpersonal contact at the individual level, reflecting individual differences. This is fruitful since a person can for example self-report interpersonal contact with other community members even when the degree of interpersonal contact within the community is low overall (Volker et al., 2006). Nonetheless, future studies could examine community identification and interpersonal contact both at the individual and at the community level and examine whether they would have a similar effect. Furthermore, interpersonal contact might be high within certain subgroups within the community, yet this does not necessarily imply high levels of participation beyond these groups, inhibiting the spread of sustainable energy behavior throughout the community at large (Granovetter, 1983; Gould, 1993). Thus, future research could account for community-level embeddedness, studying the extent of personal contact with different members within social networks in these communities to provide additional insights.

### Practical Implications

The findings in this paper emphasize that community factors, and particularly community involvement, are related to people’s initiative involvement. To motivate such involvement, it seems key to convey the benefits of the CEI for the community, in addition to appealing to people’s personal sustainable energy motivations. In particular, people may not only become involved in CEIs because they are motivated for sustainable energy behavior. In fact, we found people not more likely to attend a meeting when they found engaging in sustainable energy behavior important, suggesting people are particularly motivated to become involved in a CEI because they are involved in their community. Thus, communicating the communal aspects of CEIs, such as these initiatives enabling community members to meet and connect may be an effective way of motivating community members to become involved. Specifically, our findings suggest CEIs could emphasize people’s attachment to their community (capitalizing on their initiative identification) as well as making use of interpersonal contacts among community members in order to promote initiative involvement.

Related to this, our finding of the role of interpersonal contact points to possible ways to identify communities that are particularly promising for successful CEI initiatives. Broadly, this would entail targeting communities with many interpersonal contacts between their inhabitants next to focusing on communities with a relatively high interest in sustainable energy. In doing so, practitioners should ensure not to increase (social) inequalities between different types of local communities but allow all communities to realize the potential social and environmental benefits CEIs may offer.

In conclusion, our findings show that research into different community factors is an important new avenue for improving both scientific understanding of, and policies supporting, involvement in CEIs. The findings of our study indicate that people’s community involvement is uniquely related to their involvement in a CEI, next to the extent to which they are personally motivated to engage in sustainable energy behavior. Importantly, we disentangle two aspects of being involved in one’s community: identification with the community and interpersonal contacts with others in the community, and both seem to play a role in motivating initiative involvement. These insights suggest policy makers and practitioners should give more importance to the role these community factors could play in promoting involvement in community energy initiatives.

## Data Availability Statement

The raw data supporting the conclusions of this article will be made available by the authors, without undue reservation.

## Ethics Statement

The studies involving human participants were reviewed and approved by the Heymans Institute Ethics Committee. The patients/participants provided their written informed consent to participate in this study.

## Author Contributions

FG and DS wrote the initial draft of the manuscript, conducted the analysis, designed the study, and acted as joint lead authors. FG collected the data. LJ, LS, AF, and JD provided the supervision and critical feedback on several versions of the manuscript. All authors contributed to writing the final manuscript.

## Conflict of Interest

The authors declare that the research was conducted in the absence of any commercial or financial relationships that could be construed as a potential conflict of interest.

## Publisher’s Note

All claims expressed in this article are solely those of the authors and do not necessarily represent those of their affiliated organizations, or those of the publisher, the editors and the reviewers. Any product that may be evaluated in this article, or claim that may be made by its manufacturer, is not guaranteed or endorsed by the publisher.

## Footnotes

1 We follow an adjusted description of the methodology and relevant measures from this larger study (see Goedkoop, 2021).

2 In small villages, “my neighborhood” was replaced with “my village”.

3 For further detail on these two components of initiative involvement, see Supplementary Appendix 4 for a separate analysis of contributing financially and volunteering.

4 We used a partial proportional odds model using the VGLM function from the VGAM package in R. Note that the natural logarithm of the odds of being in a category higher than *j*, instead of lower, which is usually the case in proportional odds models, is estimated here. Thus, while in a proportional odds model positive estimates are related to a higher likelihood of being in a higher category of the outcome variable, in this case the estimates are actually reverse, due to this difference in model specification. Yet, because the estimates remain exactly the same, for reasons of interpretation, results are shown in a similar way as in a proportional odds model, where a positive effect means an increase in the probability of willingness to participate (Yee, 2010, 2015).

5 Since people’s education level and gender, have been shown to be related to pro-environmental behavior (Gifford and Nilsson, 2014), we additionally controlled for these socio-demographic variables. For ease of interpretation we display results including additional control variables in Supplementary Appendix 3.

6 Unless explicitly noted otherwise, effects we report as being supported by the data are statistically significant at a level of *p* < 0.05.

7 Indeed, when looking at the difference scores in our sample between personal sustainable energy motivation and community sustainable energy motivation, we find that the more people identify with the community, the smaller their difference score (*r* = −0.38; *p* < 0.001).
