# Supplementary material for: The Role of Community in Understanding Involvement in Community Energy Initiatives
Source: Front Psychol. 2022 Feb 9;12:775752. doi: 10.3389/fpsyg.2021.775752 (PMC8864767; doi:10.3389/fpsyg.2021.775752)
Supplement: Supplementary file 1 [file Data_Sheet_1.pdf]

Supplementary Material

Contents

1 Appendix 1. Chi square test between initiative meeting attendance and willingness to participate 2

2 Appendix 2. Plots of interaction effects between community sustainable energy motivation and identification with the community for willingness to participate 3

3 Appendix 3. Models including control variables 4

4 Appendix 4. Partial odds models of willingness to participate as volunteer or financially invest separately 6

1 Appendix 1. Chi square test between initiative meeting attendance and willingness to participate

Table 1. *Chi square test between initiative meeting attendance and willingness to participate*

	Willingness to participate		
Initiative meeting attendance	No	Maybe	Yes
No	25.0%	53.0%	3.6%
Yes	3.6 %	11.5%	3.3%

χ^2 (2, N = 439) = 18.52, $p < .001$.

2 Appendix 2. Plots of interaction effects between community sustainable energy motivation and identification with the community for willingness to participate

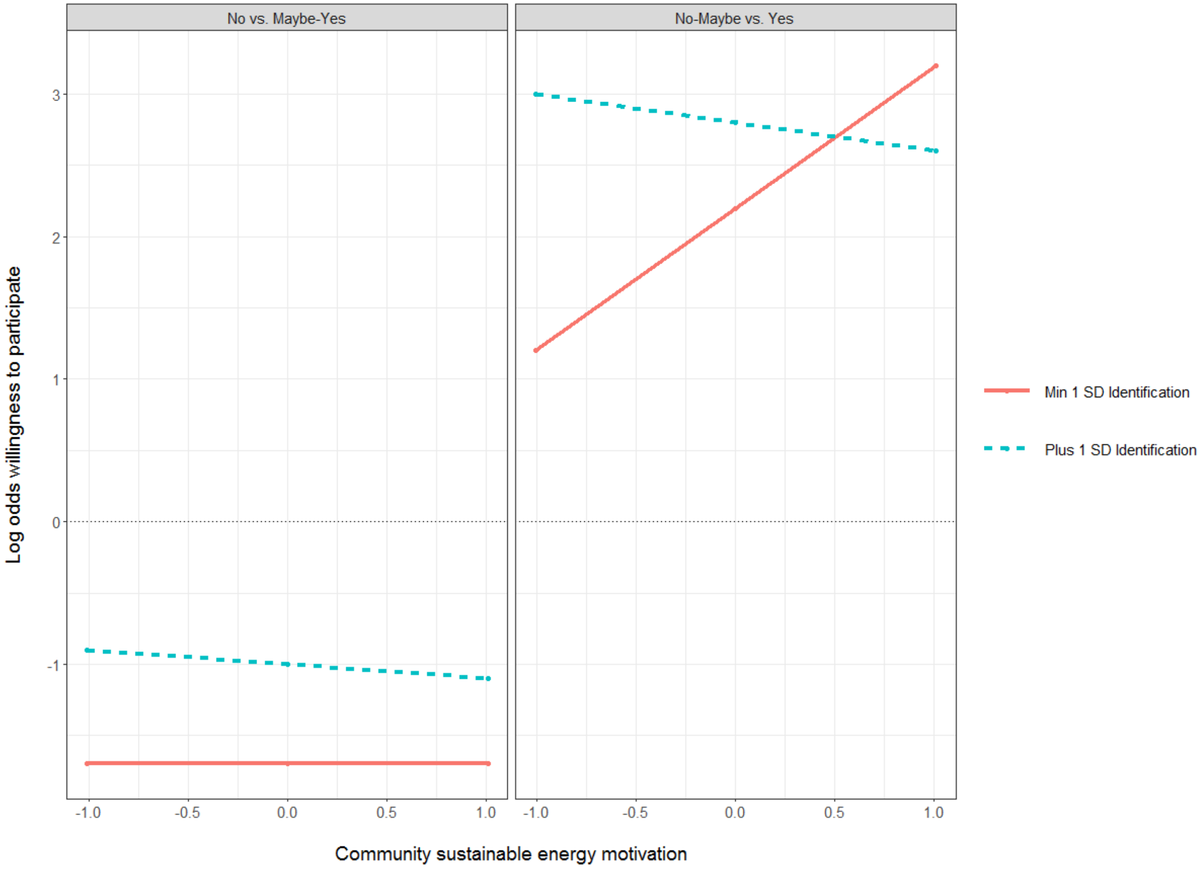

Figure 1. Simple slopes plots of the interaction effect between community sustainable energy motivation and identification with the community for willingness to participate [1]

---

[1] Note that in our sample 67 respondents scored one SD below the mean or lower in the data and 60 respondents scored one SD above or higher, indicating a relatively even distribution across levels of community identification.

3 Appendix 3. Models including control variables

Table 2. *Partial proportional odds model of willingness to participate in a CEI. Unstandardized coefficients and standard errors*.

	Step 1		Step 2		Step 3a		Step 3b		Step 4a		Step 4b		Step 3c		Step 4c	
	Estimate	*p*	Estimate	*p*	Estimate	*p*	Estimate	*p*	Estimate	*p*	Estimate	*p*	Estimate	*p*	Estimate	*p*
Threshold 1	–1.28*	.017	–1.17*	.028	–1.28*	.016	–1.22*	.022	–1.31*	.015	–1.22*	.022	–1.23*	.020	–1.24*	.021
Threshold 2	2.49***	< .001	2.65***	< .001	2.57***	< .001	2.67***	< .001	2.46***	< .001	2.67***	< .001	2.66***	< .001	2.61***	< .001
Personal motivation	0.37***	<.001	0.32**	.002	0.34**	.002	0.32**	.003	0.36**	.001	0.34**	.002	0.35**	.002	0.36**	.001
Community motivation																
[No vs. Maybe-Yes]			0.24*	.037	0.05	.689	0.21	.072	–0.04	.775	0.19	.123	0.06	.653	0.04	.768
[No-Maybe vs. Yes]			0.28*	.012	0.23	.273	0.16	.422	0.41	.062	0.28	.194	0.21	.341	0.45*	.049
Identification with the com.																
[No vs. Maybe-Yes]					0.31**	.003			0.30**	.004			0.36**	.004	0.34**	.005
[No-Maybe vs. Yes]					0.09	.633			0.18	.335			–0.006	.632	0.02	.934
Interpersonal contact																
[No vs. Maybe-Yes]							0.13	.366			0.13	.373	–0.10	.558	–0.10	.536
[No-Maybe vs. Yes]							0.49*	.049			0.64*	.014	0.53	.050	0.66*	.027
Community motivation × Identification with the com.																
[No vs. Maybe-Yes]									–0.03	.574					–0.03	.765
[No-Maybe vs. Yes]									–0.43*	.013					–0.41*	.038
Community motivation × Interpersonal contact																
[No vs. Maybe-Yes]											–0.06	.585			–0.03	.822
[No-Maybe vs. Yes]											–0.41	.067			–0.18	.532
Female [male = ref]	–0.94***	< .001	–0.94***	< .001	–0.92***	< .001	–0.96***	< .001	–0.93***	< .001	–0.98***	< .001	–0.93***	< .001	–0.94***	< .001
Education [low= ref]																
Medium	0.57	.075	0.68*	.039	0.76*	.022	0.68*	.039	0.73*	.030	0.66*	.045	0.75*	.023	0.74*	.029
High	0.02	.964	0.07	.818	0.10	.756	0.10	.748	0.08	.819	0.10	.757	0.11	.712	0.11	.746
AIC	657.63		655.80		651.01		655.60		649.04		655.88		648.87		651.26	

*p < .05. **p < .01. ***p < .001. Note: N = 439. All results are shown in log odds and controlled for community fixed effects.

Table 3. *Binomial logistic regression of initiative meeting attendance. Unstandardized coefficients and standard errors.*

	Step 1		Step 2		Step 3a		Step 3b		Step 4a		Step 4b		Step 3c		Step 4c	
	Estimate	*p*	Estimate	*p*	Estimate	*p*	Estimate	*p*	Estimate	*p*	Estimate	*p*	Estimate	*p*	Estimate	*p*
Intercept	–1.76*	.014	–1.97**	.008	–1.82*	.016	–1.98**	.009	–1.84*	.015	–1.98**	.009	–1.86*	.014	–1.84*	.016
Personal motivation	0.25	.105	0.19	.228	0.17	.293	0.17	.292	0.16	.326	0.15	.363	0.17	.317	0.15	.363
Community motivation			0.43**	.003	0.22	.206	0.32*	.039	0.21	.210	0.31*	.041	0.21	.227	0.21	.235
Identification with the com.					0.41**	.005			0.39**	.005			0.26	.089	0.30	.076
Interpersonal contact							0.56**	.003			0.54**	.005	0.40	.056	0.36	.102
Community motivation × Identification with the com.									–0.02	.827					–0.05	.683
Community motivation × Interpersonal contact											0.13	.387			0.19	.340
Female [male = ref]	–1.02**	.001	–1.07**	.001	–1.04**	.002	–1.13***	< .001	–1.04**	.001	–1.11***	< .001	–1.10**	.001	–1.08**	.001
Education [low= ref]																
Medium	1.12*	.032	1.25*	.020	1.40*	.010	1.28*	.018	1.41*	.010	1.29*	.017	1.36*	.012	1.35*	.013
High	0.64	.236	0.67	.225	0.74	.183	0.73	.189	0.75	.179	0.73	.189	0.76	.172	0.74	.187
AIC	352.15		345.09		338.54		337.91		340.50		339.19		336.91		339.92	

*$p < .05$. **$p < .01$. ***$p < .001$. Note: N=439. All results are shown in log odds and controlled for community fixed effects.

4 Appendix 4. Partial odds models of willingness to participate as volunteer or financially invest separately

Table 4. *Partial proportional odds model of willingness to volunteer in a CEI. Unstandardized coefficients and standard errors*.

	Step 1		Step 2		Step 3a		Step 3b		Step 4a		Step 4b		Step 3c		Step 4c	
	Estimate	*p*	Estimate	*p*	Estimate	*p*	Estimate	*p*	Estimate	*p*	Estimate	*p*	Estimate	*p*	Estimate	*p*
Threshold 1	0.19	.723	–0.25	.645	–0.18	.749	–0.30	.604	–0.16	.773	–0.30	.596	–0.24	.669	–0.22	.697
Threshold 2	3.63***	< .001	3.75***	< .001	3.67***	< .001	3.88***	< .001	3.61***	< .001	3.92***	< .001	3.81***	< .001	3.81***	< .001
Personal motivation	0.41***	< .001	0.38***	< .001	0.39***	< .001	0.37***	< .001	0.39***	< .001	0.37***	< .001	0.38***	< .001	0.39***	< .001
Community motivation																
[No vs. Maybe-Yes]			0.12	.254	0.01	.930	0.04	.714	–0.02	.894	0.05	.649	0.03	.798	0.02	.850
[No-Maybe vs. Yes]			0.35	.142	0.33	.230	0.19	.475	0.36	.204	0.18	.493	0.24	.399	0.31	.307
Identification with the com.					0.22*	.024			0.22*	.026			0.15	.166	0.14	.191
[No vs. Maybe-Yes]					0.04	.878			0.06	.816			0.14	.586	0.12	.639
[No-Maybe vs. Yes]																
Interpersonal contact																
[No vs. Maybe-Yes]							0.41**	.005			0.41**	.005	0.34*	.027	0.33*	.034
[No-Maybe vs. Yes]							0.63*	.041			0.58	.076	0.68*	.036	0.54	.139
Community motivation × Identification with the com.									–0.02	.741					–0.07	.399
[No vs. Maybe-Yes]									–0.13	.513					–0.32	.147
[No-Maybe vs. Yes]																
Community motivation × Interpersonal contact																
[No vs. Maybe-Yes]											0.05	.651			0.11	.381
[No-Maybe vs. Yes]											–0.13	.583			0.41	.160
AIC	635.94		636.94		635.71		630.84		639.17		634.41		632.36		637.47	

$^{*}p$ < .05. $^{**}p$ < .01. $^{***}p$ < .001. Note: N=439. All results are shown in log odds and controlled for community fixed effects, gender, and education.

Table 5. *Partial proportional odds model of willingness to financially invest in a CEI. Unstandardized coefficients and standard errors*.

	Step 1		Step 2		Step 3a		Step 3b		Step 4a		Step 4b		Step 3c		Step 4c	
	Estimate	*p*	Estimate	*p*	Estimate	*p*	Estimate	*p*	Estimate	*p*	Estimate	*p*	Estimate	*p*	Estimate	*p*
Threshold 1	–1.03	.051	–.93	.072	–1.04*	.047	–.95	.068	–1.20*	.024	–1.02	.052	–.99	.056	–1.14**	.003
Threshold 2	2.65***	< .001	2.78***	< .001	2.67***	< .001	2.78***	< .001	2.54***	< .001	2.73***	< .001	2.74***	< .001	2.63***	< .001
Personal motivation	0.35**	.001	0.31**	.003	0.32**	.003	0.31**	.003	0.37***	< .001	0.33**	.002	0.32**	.002	0.38***	< .001
Community motivation																
[No vs. Maybe-Yes]			0.24*	.029	0.10	.429	0.22*	.049	0.03	.081	0.17	.144	0.10	.405	0.04	.758
[No-Maybe vs. Yes]			0.19	.315	0.12	.599	0.11	.604	0.41	.074	0.16	.460	0.09	.673	0.42	.071
Identification with the com.					0.23*	.024			0.21	.039			0.25*	.025	0.24*	.035
[No vs. Maybe-Yes]					0.14	.493			0.22	.278			0.02	.919	0.11	.627
[No-Maybe vs. Yes]																
Interpersonal contact																
[No vs. Maybe-Yes]							0.08	.585			0.06	.659	–0.09	.578	–0.10	.503
[No-Maybe vs. Yes]							0.34	.193			0.43	.101	0.32	.274	0.36	.250
Community motivation × Identification with the com.									–0.17*	.012					–0.18*	.045
[No vs. Maybe-Yes]									–0.63***	< .001					–0.66**	.002
[No-Maybe vs. Yes]																
Community motivation × Interpersonal contact																
[No vs. Maybe-Yes]											–0.17	.108			–0.02	.909
[No-Maybe vs. Yes]											–0.36	.112			–0.08	.905
AIC	668.91		667.81		666.62		670.02		655.67		669.28		668.86		661.32	

**p* < .05. ***p* < .01. ****p* < .001. Note: *N*=439. All results are shown in log odds and controlled for community fixed effects, gender, and education.
